# Sonic Sleight of Hand: Sound Induces Illusory Distortions in the Perception and Prediction of Robot Action

**DOI:** 10.1007/s12369-024-01105-5

**Published:** 2024-02-17

**Authors:** Joel Currie, Maria Elena Giannaccini, Patric Bach

**Affiliations:** 1 School of Psychology, University of Aberdeen, St Machar Drive, Aberdeen, AB24 3FX UK; 2 School of Engineering, University of Aberdeen, Elphinstone Rd, Aberdeen, AB24 3UE UK

**Keywords:** Human–robot interaction, Social robotics, Representational momentum, Movement sonification, Cue-integration, Motion perception

## Abstract

For efficient human–robot interaction, human operators need to be able to efficiently represent the robot’s movements in space and predict its next steps. However, according to frameworks of Bayesian multisensory integration, features outside the motion itself—like the sounds a robot makes while it moves—should affect how otherwise identical motions are perceived. Here, we translate an established psychophysical task from experimental psychology to a human–robot interaction context, which can measure these distortions to motion perception. In two series of preregistered studies, participants watched a humanoid robot make forward and backward reaching movements. When the robot hand suddenly disappeared, they reported its last seen location, either with the mouse cursor (Experiment 1a and 1b) or by matching it to probe stimuli in different locations (Experiment 2a and 2b). The results revealed that even small changes to the robot’s sound robustly affect participants’ visuospatial representation of its motions, so that the motion appeared to extend further in space when accompanied by slightly (100 ms) longer sounds compared to slightly shorter sounds (100 ms shorter). Moreover, these sound changes do not only affect where people currently locate the robot’s motion, but where they anticipate its future steps. These findings show that sound design is an effective medium for manipulating how people represent otherwise identical robot actions and coordinate its interactions with it. The study acts as proof of concept that psychophysical tasks provide a promising tool to measure how design parameters influence the perception and prediction of robot motion.

**Supplementary Information:**

The online version contains supplementary material available at 10.1007/s12369-024-01105-5.

## Introduction

Robots are increasingly used in close interaction with humans. In manufacturing, robots work together with humans on flexible production lines for tasks that cannot be fully automated [2, 41, 74]. Close physical human–robot interaction is important in rehabilitation [43] to support patients in everyday life and to improve their agency [55, 59], as well as in therapy and socially demanding service work [34, 94]. Robots are also used for teleoperation in environments that are not safe for human operators [68], or where domain experts are not available locally [14].

What limits productive human–robot interactions in these and other applications is that robots often behave in a way that people find unnatural [76]. Human-to-human cooperation is built on the ability to effortlessly “read” and predict the behaviour of one’s interaction partners [5, 6, 73, 82, 86]. From simple tasks like handing over tools or moving a table to more complex ones such as assembling an object together, efficient cooperation relies on the ability to represent our partner’s actions [78]. In human-to-human cooperation, these interactions are supported by sophisticated internal models that capture how other intentional (human) interaction partners behave within a given environment [5, 20, 33, 49]. These models do not only supply observers with higher-level semantic information about which action another agent is currently executing [73], but also with more fundamental visuospatial information about these actions’ lower-level kinematics, such as changes in spatial location, direction, and speed, which are crucial for guiding moment-to-moment interactions in a shared task space [61]. Importantly, observers represent these kinematic parameters in a predictive manner, capturing not only the movements’ current state but also how they will likely develop in the immediate future, so that one’s own actions can be directed not towards where the motion is now but where it will be in its next steps [5, 33, 49]. Non-biological agents like robots do not fit these human-centric internal models [91], however, making it difficult for people to understand and predict their behaviour [18, 48].

Different approaches have investigated how human understanding of robot behaviour can be improved, from making robots communicate via gestures [42], endowing them with human-like social cues such as eye-gaze [67], to giving them the ability for speech [46], with limited success [8]. Here, we investigate an important but typically neglected factor that may affect the observer’s perception of robot kinematics: the sounds that a robot produces as it moves. This can include not only the robot’s consequential sounds, such as the noise of its motors, the friction of its moving parts and the whirring of fans [83], but also supplemental intentional sounds that are designed to convey additional information, such as an emulated emotional state [80].

Testing how sound affects the perception of robot behaviour is important because human motion perception is multisensory. The visual perception of a moving stimulus is automatically integrated with information received from other senses, as well as with prior information about how the motion is likely to continue, to arrive at a statistically optimal estimate of what is observed [24, 89, 92]. Both the intentional and consequential sounds that accompany robot motions would provide a central component to these estimations. Sounds should therefore be involuntarily integrated into the perception of robot motion, and — especially when sound and motion are not well aligned—should induce illusory distortions in how the motion is represented. Identical visual motions could therefore be perceived differently depending on the features of the sounds that accompany them. Importantly, because human observers are typically not aware of this integration, they often experience it as an audiovisual illusion [1, 9, 23, 51]. Any induced changes will therefore persist over repeated exposures, unless they are counteracted by specifically designed feedback, or become noticeable through (potentially costly) action errors that human operators may make. Moreover, research suggests that such illusory changes will, if anything, increase when people’s attention is directed towards another task that they are engaged in [53], as is likely in most human-robot interactions.

So far, only a handful of studies have investigated how a robot’s sound is integrated into the perception of robot motion (see [70] for general overview of sound in HRI, [95] for review specifically of nonverbal sound). Most of these studies have measured only its impact on the evaluation of the robot’s higher-level socio-emotive features, such as its attractiveness, quality of movement and perceived safety [69], or which sound characteristics facilitate its localisation in the absence of visual input [10]. Nothing is known about how sound changes the perceptual representation of robot motion itself. Yet, as noted above, investigating such visuospatial changes is crucial because these features are what is ultimately used by human interaction partners to plan their own responses within the shared task space. Distortions to a movement’s visuospatial representation will therefore have a direct effect on the actions that human operators direct towards the robot [58, 60]. Moreover, even higher-level socio-emotive responses to robot movements likely stem in part from lower-level representations of its kinematic features (e.g., its jerkiness, smoothness, etc.), or from mismatches of motion signals in different channels (“uncanniness” [44, 75]). So far, however, no tasks are available that would enable researchers to probe how human observers visuospatially represent a robot’s motion.

The goal of the current study is to (1) develop an experimental task that can sensitively measure the predictive kinematic representations that humans derive of motions and (2) make it usable for HRI research. We will use (3) the robot’s sound as a testing bed to investigate whether these representations can be effectively measured and manipulated. We draw upon the well-established family of representational momentum-like tasks [19, 28], which ask participants to accurately localise the last seen position of briefly seen motions (e.g., with a mouse pointer, or by comparing it to “probe” stimuli presented in different locations on the screen). Findings from this task have shown that, to localize moving objects, humans do not just rely on the parameters of the motion itself but improve their estimates by drawing on all available evidence across the multisensory perceptual sphere, as well as their prior expectations about how the motion will continue, and integrate it following the principles of Bayesian computation [16, 37]. People’s perception of motion therefore does not veridically represent the visual motion input, but is (illusorily) distorted towards the motion’s expected next steps, which allows agents to compensate for inherent delays in motor control, making real-time interactions with a dynamic environment possible [25, 26, 61]. For example, when asked to accurately report disappearance points of moving objects, location judgments are (erroneously) displaced away from the objects true location towards the expected next steps of the motion sequence, and these misperceptions incorporate one’s prior knowledge of the forces acting on the object [27], intuitive knowledge of physics [21], context, and in social interactions, the goals attributed to the agent [5, 30–33, 49, 50].

Here, we use this task to probe people’s kinematic representation of robot movements, and measure how it is affected by the robot’s sound. In four studies, participants viewed brief sequences of a robot hand in side view reaching or withdrawing (see [30] for similar design in human action perception). The robot’s hand disappeared at an unpredictable point during the action, and participants were asked to accurately report its last seen location, either with their mouse cursor (Experiments 1a and 1b) or by comparing it to static probe stimuli showing the robot hand displaced forwards or backwards in time (Experiments 2a and 2b). To test whether the robot’s sound affects motion representation, we manipulated the duration of the sound that accompanied the movements, as an important ecologically valid sound component. As a complex system made up of various components, a robot’s consequential sounds will not always begin and end simultaneously, due to inherent delays and their position in the operational hierarchy. For example, a motor may be most active during the early stage of a motion, while a fan that cools it may continue to operate even after the motion has ceased. Similarly, supplemental sounds may be deployed out of sync with a robot’s motion due to computational error, or intentionally to achieve a desired outcome. If sound duration is integrated into the perception of visual motion, then changes to sound duration should affect the extent of the perceived motions. Reaches and withdrawals should therefore be reported to have travelled further along their trajectory when accompanied by a longer sound than a shorter sound [84].

Numerous manipulations of robot sounds hold promise for introducing biases in how robot actions are represented perceptually. For example, Orthmann and colleagues found that robot actions were rated faster when accompanied by sounds with higher frequency than when accompanied by sounds with a lower frequency [7]. Spatially distributed robot sounds influence participants’ ratings of animacy and agency [71]. We chose to manipulate the duration of the sound that accompanied the robot’s actions, as prior evidence from outside HRI indicated that sound duration is integrated with perceived motion, abstract shapes making the motion appear more or less pronounced depending on the duration of the sound that accompanies it [84]. Importantly, this manipulation of sound duration can be incorporated on very simple hardware, and therefore has wide application, unlike spatially distributed sounds that require a more complex setup.

Our research strategy is as follows. In Experiment 1a and the preregistered Experiment 1b, we ask participants to accurately localise the robot hand’s disappearance points using a computer mouse. These studies show indeed that sound is integrated with human perception of robot action and affects how otherwise identical motions are perceived, so that visually identical disappearance points are localised further along the motion path when accompanied by sounds of a longer duration and not as far when the sounds terminated earlier. Importantly, mouse responses rely on the same visuospatial motor maps that people use to coordinate the movements of their own limbs within a dynamic environment [36, 57, 58] and can therefore serve as a proxy for the visuospatial processes guiding dynamic human–robot-interaction. However, while they robustly capture the effect of sound on spatial localisation, they are subject to various biases that render them unable to precisely measure how perceptual representations more generally relate to the objective motion that was perceived, specifically whether the representation of robot movement is predictive or lags behind the perceived motion. To provide this crucial test, in Experiments 2a and 2b we ask participants to make their motion disappearance judgments not with a mouse cursor, but by comparing them to static “probe” stimuli presented after. The non-spatial nature of these responses eliminates the confounding biases and shows for the first time that human representation of robot is inherently predictive, capturing not only its last seen location but being enriched by expectations of how it will develop in the future.

## Experiments 1a and 1b

Hypothesis: We predict that participants’ perception of where a robot’s moving hand disappeared (measured using participant mouse response data on the x-axis) will be biased by the sound that accompanies it, so that the hand motion will appear to extend further along the action trajectory when the sound accompanying it continues for longer than the motion ($+100$ ms positive auditory offset), relative to when the sound ends sooner than the motion (negative offset $-100$ ms).

### Methodology

#### Participants

Participants (Experiment 1a: 51 participants, 35 women including trans women, 16 men including trans men, mean age 21.2, SD (standard deviation) = 3.32, 41 right-handed; Experiment 1b: 51 participants, 32 men including trans men, 19 women including trans women, mean age 32.6 years, SD = 10.13, 48 right-handed), were recruited using the University of Aberdeen’s research participation scheme (Experiment 1a) and Testable Minds (Experiment 1b). They gave electronic informed consent as part of the experiment briefing and were reimbursed with course credits or $\pounds$ 4.70. Each experiment took approximately half an hour.

In Experiment 1a, the final sample of 44 participants provides.80 power to detect effect sizes of *d* (Cohen’s d) $$=.43 (n_{p}^{2} $$ Partial eta squared $$ = 0.159)$$. For Experiment 1b, the final sample of 42 gives us.80 power to detect effects of at least $$d =.44 (n_{p}^{2} = 0.165)$$. Prior studies investigating multisensory integration in biological motion outside HRI [30–32] revealed that effect sizes in similar paradigms are consistently of this size or larger ($$d =.52$$ to $${d} = 1.23$$).

#### Apparatus

Visual stimuli were filmed on a Canon m50 mirrorless camera and edited using Lightworks and VLMC. Audio stimuli were created using samples from the sound repository freesound which were edited using Audacity [4] and MATLAB [47]. The experiment was programmed using the Inquisit platform [54] in an online format. Participants used their own personal devices to complete the experiment. Included devices were personal computers with a mouse and keyboard. Participants with mobile devices were asked not to participate and excluded if they did. Participants were asked to wear headphones during the experiment, and their ability to hear the sounds played was self-verified on the basis of test sounds (simple beeps) at the start of the experiment.

**Fig. 1:**
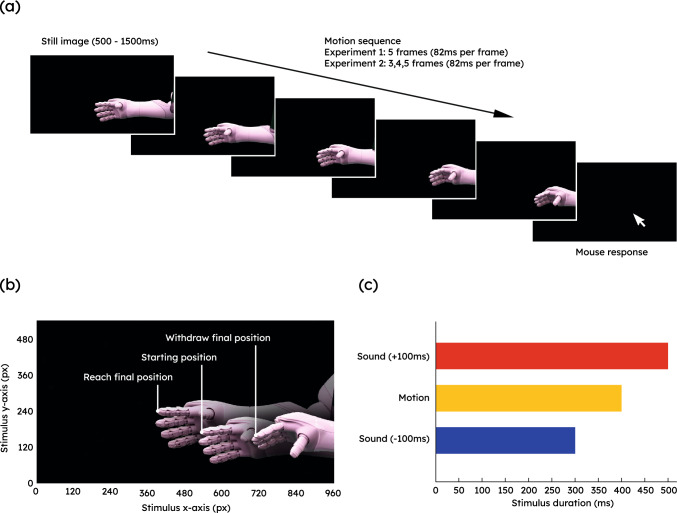
**a** Trial sequence example and relative lengths of presented visual and auditory stimulus. This trial sequence shows a withdraw. During the response stimulus the participant uses their mouse to indicate the last perceived location of the robot’s index finger. **b** Starting position and final Reach and Withdraw positions from a 5-frame motion. **c** Relative motion and auditory offsets

#### Stimuli

**Visual Stimuli**: Visual stimuli were derived from video recordings showing the side profile of the humanoid robot Pepper (Aldebaran) [62], completing a reaching (leftward), or a withdrawing (rightward) action. The grasping trajectory was designed to emulate a human agent reaching towards, and then grasping, an object. To reduce unnecessary uncertainty for participants unfamiliar with robot kinematics the designed grasp/withdraw trajectory was biologically inspired. As humans sample human motion daily, they have an inherent robust model of human motion [3, 64]. Due to this it was important for the robot’s morphology to match the human-like trajectory profile. Of the robots available to our lab, the Pepper robot best suited this task. Background details in the video were replaced with a uniform black background using the video editing software Lightworks.

Experiment 1a: All stimuli were derived from an initial set of video recordings of three reach actions, starting on the right side of the screen, reaching towards the left, consisting of 32 frames in total. Frame 1 therefore shows the robot hand in its most backwards (close to the body) position while frame 32 shows the robot arm in its most extended position. Six sequences of motion were generated from this original set, each 5 frames long (6 including the initial still frame), three of them showing reaches and three showing withdrawals. The action sequences started with a neutral frame, which was randomly chosen between three alternatives (frame 13, 14, 16), showing a medium extension of the arm. Reaches and withdrawals were generated by either stepping forwards (e.g., frame 15–17–19–21–23) for reaches, and backwards (e.g., frame 11–9–7–5–3) for withdrawals through the frames of the original video stimulus sequences. Due to the three different starting positions, each action sequence could terminate in different locations in space, either closer to the centre of the screen, in a medium distance from the centre, or further out in the periphery. Each frame of the sequence was displayed for 82 ms, and immediately replaced with the next frame without gap.

The robot actions were presented as a rapid succession of static frames instead of video because this mode of presentation affords precise control of the timing of onset and offset of each frame and ensures that stimuli are not contaminated by compression/video artifacts on different platforms. Importantly, the presentation of each frame in rapid succession without any temporal gaps matches the usual frame rate in animation (12 fps $\approx 82$ ms) and induces the full perception of motion ("Apparent motion", [90]), while guarding against a contribution of smooth-pursuit eye movements, which could otherwise contaminate results in representational-momentum-like designs [36]. In additional work we have confirmed that results do not differ when a smoother (double frame rate) mode of presentation is used.

Experiment 1b: As in Experiment 1a, three reach and three withdrawal sequences were generated from the videos of the original reach sequences. In contrast to Experiment 1a, the starting position of the action sequence remained identical across sequences (frame 14), while the extent of the motion could vary between 3, 4, or 5 frames (representing a motion covering a short distance, an intermediate distance or the longest distance, respectively). This does not include the initially presented still starting frame. As before, reaches were created by stepping forward through the initial video sequence and withdrawals by stepping backwards through them. Due to the different sequence lengths, each action sequence could again terminate in different locations in space, either closer to the centre of the screen, in a medium distance from the centre, or further in the periphery. Each frame within the sequence was displayed for 82 ms and immediately replaced with the next frame without gap.

**Auditory Stimuli** We chose to design our own audio stimuli using samples from the collaborative sound repository freesound instead of using recordings from the Pepper robot, as this was dominated by the clicking and squeaking of the housing for which duration was difficult to manipulate. To this end, we used two recorded samples of consumer motors, but increased the amplitude of low frequencies to give the impression of a robot with larger mass [7] such as Pepper. Dead zones in the audio were removed. The resulting audio stimuli provided an ideal testing bed for the sound offset manipulation, providing realistic continuous sound with a clear onset and offset. Moreover, while intentionally designed to supplement the robot’s movements, they are based on features associated with the robot’s consequential (motor) sounds, supporting the formation of a causal link between robot movement and accompanying sound [15].

Experiment 1a: Two versions of the audio stimuli were generated, with durations of 310 ms and 510 ms, representing an offset of ($-100$ ms and $+100$ ms) relative to the visual motion sequences. These offset values were chosen from literature outside the HRI context [84], as these sufficed to induce measurable shifts in perceptual judgments while being not readily detectable by participants.

Experiment 1b: Six versions of the designed audio were generated. Three represented a 100 ms offset after the termination of the motion sequences, and three a 100 ms offset before their offset. This resulted in durations of 146 ms and 346 ms for the 3-frame sequence, 228 ms and 428 ms for the 4-frame sequence, and 310 ms and 510 ms for the 5-frame sequence.

#### Procedure

The participants first gave informed consent and received experimental instruction. They then proceeded to complete eight training trials, identical to the experimental trials. Training trials could be repeated if the participants felt they did not fully understand the task. Both Experiment 1a and 1b consisted of 120 trials, presented in 2 blocks of 60 trials each, with conditions following in pseudo-randomized order, counterbalanced so that each combination of the three sequence lengths/starting positions, two action directions, and two sound durations (100 ms longer or shorter than the movement durations) were repeated an equal number of times. In Experiment 1b, after the 120 experimental trials were completed, participants were asked to make a (free text) guess of the experimental hypothesis, to test whether the effects obtained can be explained by participants guessing the experimental hypothesis and adapting their behaviour accordingly (i.e., demand effects). Additionally, participants were asked questions probing their awareness of a change in the two sound conditions during the experiment (for details see in the supplementary information, testing for demand effects). The experiment lasted about 30 min in total.

At the beginning of each trial (one robot action), participants were presented with a static image of the first frame of the movement. Following a randomly generated delay between 1000 and 2500 ms, the action sequence (reach or withdrawal) and auditory stimuli were presented. Action sequence and auditory stimuli always began synchronously after the random interval so that participants could form a causal relationship between the sound they were hearing and the robot hand moving [15], ensuring that participants believed the sound they heard came from the robot’s motion [84]. The sequences stepped forward (for reaches) or backward (for withdrawals) through the stimulus sequence in five frames (Experiment 1a) or three, four or five frames in Experiment 1b. The last frame was immediately replaced by a black screen. The auditory stimuli stopped playing either 100 ms before the action sequence offset, or 100 ms after. The participants were asked to use their mouse and click to accurately indicate the last seen location of the robot’s index finger. They had five seconds maximum to respond. The next trial started after they clicked a green marker in the centre of the screen, so that their mouse was centred for the start of the next trial.

#### Analysis

To measure the localisation error in participant responses, the displacement between the real coordinates of the robot’s index finger ($x_{t}$, $y_{t}$), and the mouse response coordinates of participants ($x_{r}$, $y_{r}$) were calculated. Since participants completed the experiment on their own personal devices, device resolutions were captured ($x_{dr}$, $y_{dr}$) and used to scale participants’ individual responses to a universal pixel size ($1920 \times 1080$). Localisation error on the x-axis and y-axis are calculated using Eqs. 1 and 2, respectively.

1 $$\begin{aligned} e_{x}= & {} \left( \frac{1920}{x_{dr}} x_{r}\right) - x_t \end{aligned}$$

2 $$\begin{aligned} e_{y}= & {} \left( \frac{1080}{y_{dr}} y_{r}\right) - y_t \end{aligned}$$

Zero values on both axes indicate a perfect match of presented and reported disappearance points. Positive values of $e_{x}$ denote a rightward shift in responses relative to the actual final position of the robot’s hand, and negative values represent leftward responses. Positive and negative values of $e_{y}$ represent an upwards and downwards shift in responses, respectively.

The dataset was pre-processed and analysed using the statistical computing language R, Version 4.2.2 [65]. This included the exclusion of participants and trials (Sect. 2.1.6), and calculating the standardized localisation error for individual participants for each experimental condition.

Statistical analysis was completed using the function ‘ezANOVA’ from the package ‘ez’ [40] for the standardized localisation error, indicating the difference between the real location of the robot’s index finger’s disappearance point and the location the participants identified with their mouse click. Localisation error on the x-axis ($e_{x}$) was measured as the displacement between participants’ response coordinate ($x_{r}$), and the actual termination x-axis coordinate of the robot’s hand ($x_{t}$), and analogously for the y-axis.

#### Exclusion Criteria

Participants were excluded if they used a mobile device without mouse. This was the case for four participants in Experiment 1a, and five in Experiment 1b. Additionally, participants with an aggregate mean localisation error (displacement between target stimulus and participants response across all conditions) greater than 10% of displayed stimulus size were excluded. From Experiment 1a three participants were excluded based on this criterion, and one from Experiment 1b. In Experiment 1b, three participants were excluded because they consistently responded after the allocated response time interval of 5 s.

In Experiment 1a, trials with localisation error greater than 3 SD from the median were excluded. As preregistered in Experiment 1b, trials with localisation error greater than 3 SD from the median were excluded, as well as trials with response times shorter than 200 ms, or longer than 3 SD from the sample median ($\approx$2.2%).

### Results: Experiments 1a and 1b

Each participant’s mean $e_{x}$ localisation errors were entered into $2 \times 2 \times 3$ repeated measures analyses of variance (ANOVA), with factors Action Direction (Reach vs. Withdrawal), Sound ($-100$ ms vs. $+100$ ms) and End Position of motion termination relative to the centre of the participant’s screen on the x-axis (Centre, Middle, Outer). Data was analysed using the same ANOVA model for both Experiment 1a and 1b.

**Fig. 2:**
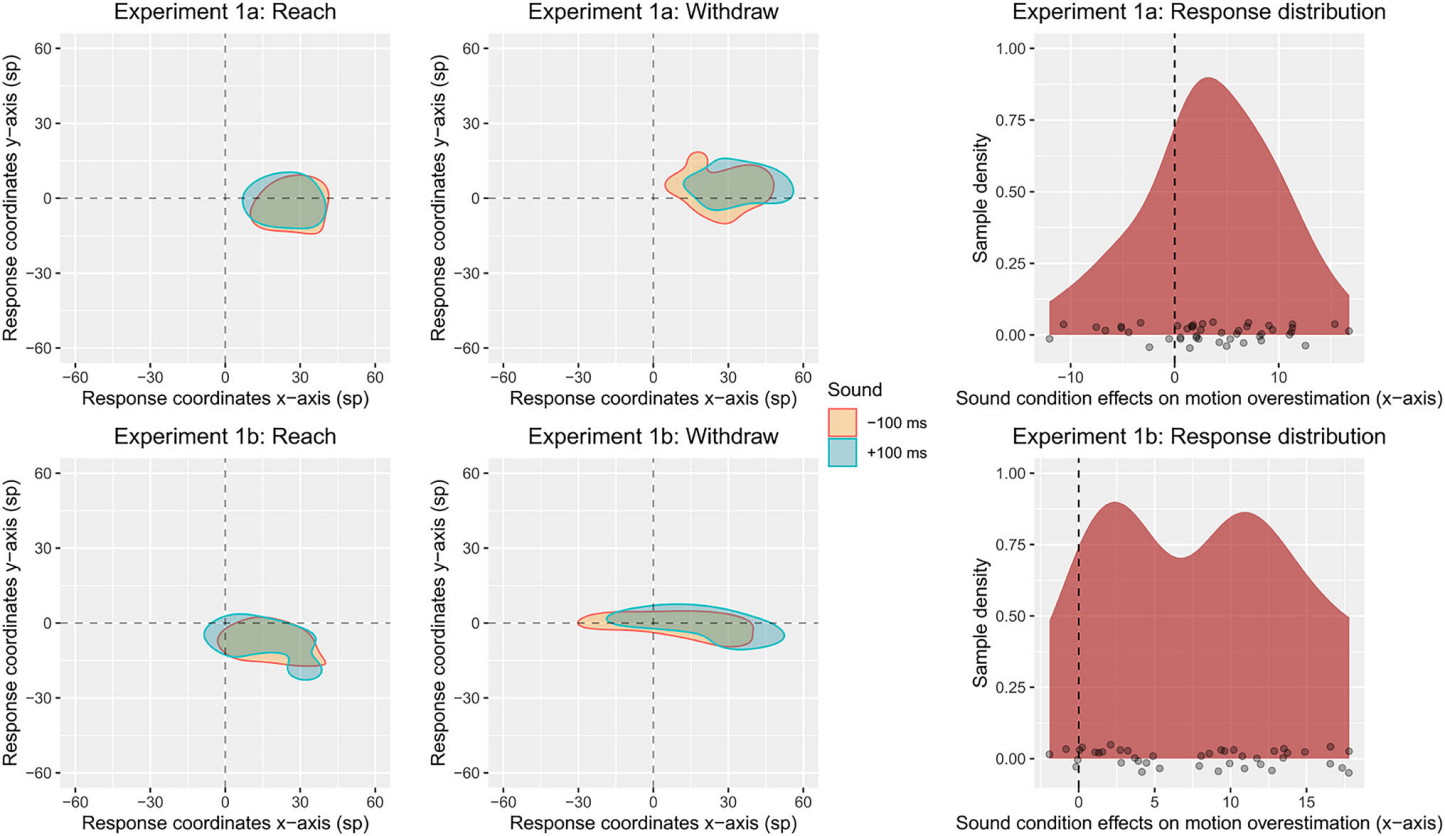
2D kernel density estimation for spatial distribution of response coordinates expressed as the difference between the real final coordinate of the robot’s index finger and participants’ response coordinate on the x-axis and y-axis. Response coordinates are shown in universal Scaled Pixels (SP). The (0, 0) coordinate represents real final position on any given trial (0 SP difference on each axis). The (0, 0) point for reaches has been placed above that for withdrawals as the real final position for reaches was above that of withdrawals. On the right, the extent to which each participant was affected by the sound manipulation is represented by dots, with their location on the x axis showing how much, in standardized pixels, the longer and shorter sounds shifted the motion localisation. The cloud represents the overall sample distribution. The dotted line represents the zero point (no difference in localisation responses between the two sound conditions)

Our predictions primarily concern the localisation errors on the x-axis, as the majority of the robot’s grasp motion is a translation on the x-axis, with limited variance on the y-axis (see Fig. 1). Our main hypothesis is tested by the interaction of Sound and Action Direction, indicating that longer sounds bias responses more strongly leftwards for (leftwards moving) reaches and more rightwards for (rightwards moving) withdrawals than shorter sounds. While not directly relevant to our hypotheses, and statistically independent from it, pilot work with this paradigm has also revealed an interaction between Action Direction and End Position. This reflects an increasing “pull” of participants’ location judgments towards the centre of the screen the more peripherally the motions terminate, in line with the well-known tendency for visual judgments to stabilize towards average disappearance locations across trials [45, 63].

As we have no further predictions, all other main effects and interactions should be treated as incidental unless meeting a (Bonferroni-adjusted) alpha threshold of.006 to account for hidden multiplicity in an ANOVA. [13]. Analysis of the y-axis data is presented in the Supplementary Information (see Experiment 1, y-axis results), and generally replicates all findings on the x-axis.

#### Experiment 1a

Analysis of participants’ localisation errors revealed the predicted two-way interaction of Action Direction and Sound ($F(1, 44) = 12.1, p =.002, n^{2}_{p} = 0.216)$. As can be seen in Fig. 2, people reported the disappearance point of reaches (leftward motions) more leftward when the robot’s sound extended beyond the motion than when it terminated before it, and more rightwards for withdrawals (rightward motions). As hypothesised, longer sounds therefore bias participants’ responses in the direction of the robot’s motion more strongly than shorter sounds, even though exactly the same visual motions were shown.

The ANOVA model also indicated a significant intercept $(F(1, 44) = 136, p <.001, n_{p}^{2} = 0.75)$. As apparent from Fig. 2, participants’ localisation errors were generally positive, indicating that they reported the disappearance point of the robot’s index finger systematically more rightwards than it really was. This rightwards bias is statistically independent of our hypothesis tests and common for stimuli that extend rightwards such as ours, as localisation responses are typically attracted by the stimulus’ centre of mass (e.g., the middle of the hand, [11, 30, 33, 49, 50]).

Finally, the expected interaction between Action Direction and End Position was found ($F(2, 88) = 50.72, p <.001, n^{2}_{p} =.61$), as well as a main effect of End Position ($F(2, 88) = 9.64, p <.001, n^{2}_{p} = 0.179$). These findings reflect the predicted general bias of mouse localisations towards the perceptual history, which average towards the centre of the screen [45, 63]. Our ANOVA models indicated no further main effects or interactions that met the adjusted threshold (all $F < 1.21, p >.328$).

#### Experiment 1b

Experiment 1b fully replicated the results of Experiment 1a. The analysis revealed, first, the predicted (and preregistered) two-way interaction of Action Direction and Sound, $F(1,41) = 71.9, p <.001, n^{2}_{p} =.637$. As in Experiment 1a, people reported the disappearance of leftward going reaches more leftwards when the sound extended beyond the motion than when it terminated before it, and more rightwards for withdrawals (Fig. 2, lower panel).

Like in Experiment 1a, our model also replicated the general biases affecting mouse localisation responses, which are independent of our hypotheses. It revealed a significant intercept, $F(1,41) = 17.9, p <.001, n_{p}^{2} = 0.304$, replicating the general rightwards bias of localisation responses towards the robot hand’s centre of mass. Moreover, it replicated the general bias of mouse responses towards the centre of the screen, as indicated by an interaction between Action Direction and End Position, $F(1,82) = 5.79, p =.004, n^{2}_{p} = 0.124$ as well as a main effect of Action Direction ($F(1, 41) = 17.9, p <.001, n^{2}_{p} = 0.304)$. Thus, as in Experiment 1a, participants’ localisations did not cover the whole of the motion but were biased centrally, and this inward bias increased the further outwards the motion terminated.

Our ANOVA models indicated no further main effects or interactions that met the adjusted threshold (all $Fs < 4.83, p > 0.011$), with the exception of a main effect of Sound $F(1, 41) = 12.2, p =.002, n_{p}^{2} = 0.231$, representing that participants responded further rightwards when presented with longer sounds, as opposed to shorter sounds, which is independent of our hypotheses. It is most likely due to the fact that the effect of sound offset on motion over-estimation was more pronounced for rightwards moving reaches (See Fig. 2, lower panel), therefore creating an overall bias towards the right.

#### Between-Experiment Comparison

In an exploratory analysis, we verified whether the motion localisation biases that were induced by the sound duration manipulation were larger in Experiment 1b than in Experiment 1a, due to the increased uncertainty about the end-positions and timing of the motion. We therefore ran the same ANOVA model on the pooled data of Experiment 1a and 1b, with Experiment (1a vs. 1b) added as an additional between-participants factor. This indeed revealed a three-way interaction between Action Direction, Sound and Experiment, $F(1, 85) = 10.64, p =.002, n^{2}_{p} =.111$), revealing that the sound had a larger effect on the perceived motion extent in Experiment 1b than Experiment 1a.

## Experiments 2a and 2b

Experiments 1a and 1b provided the first evidence that the sound emitted by a robot influences how people represent the low-level visuospatial characteristics of its motion. When participants were asked to localise the visually perceived disappearance point of briefly presented robot hand movements, their judgements were systematically influenced by the sounds it produced, so that the motions appeared to extend further into space when accompanied by a longer sound and not as far when accompanied by a shorter sound, particularly if the motion endpoints could be less reliably predicted in Experiment 1b than in Experiment 1a. Please note that these results were obtained even though we did not ask participants to make a judgments where they assumed the robot motion to have terminated after it had disappeared, or to predicts its next step, but to accurately report the hand’s last seen location. Our findings therefore indicate that the sound manipulation did not affect higher-order judgments of assumed motion endpoints, but the more fundamental visuospatial representation of the motions itself, similar to other illusory changes induced by the integration of vision and sound [1, 9, 23, 51].

The mouse movements used for spatial localization are assumed to draw upon similar visuospatial motor maps [36, 57, 58] that are used to coordinate the movements of one’s limbs within a dynamic environment and therefore capture processes during robot–human interaction within a share task space. However, localisation responses are subject to well-known biases that we also observe here, such as a rightwards bias towards the hand’s centre of mass [11, 30, 33, 49, 50], and a bias towards the middle of the screen, the starting point of the mouse pointer [45, 63] and the average location of judgments made before [17]. While these biases are independent of our hypotheses, they make it impossible to assess how participants’ representation of the observed motions more generally relates to their actual kinematics. A classic finding is that the human representation of observed motion is predictive, so that people misperceive motions displaced into the future, extrapolated towards the motion’s expected next steps [19, 24, 26]. These predictive displacements are assumed to be a major component of people’s ability to interact with a dynamic (social) environment [5, 77], compensating for delays in motor control [57, 58, 60, 61] and allowing our own responses to be planned towards where actions will terminate in the future rather than where they are right now.

To probe this predictive representation of observed robot motion, Experiment 2a and 2b changed how the influence of sounds on motion perception was measured. Participants saw and heard the same stimuli as in Experiment 1b. However, now, they judged the motion disappearance points not by moving the mouse, but by comparing the hand’s last seen location to a static “probe” comparison image, which was presented shortly after the motion disappeared and which showed the robot’s hand either further along the motion sequence than it really was (either $+1$ or $+3$ frames forwards) or not as far (either $-1$ or $-3$ frames backwards). Participants simply indicated, through a press of a button, whether the presented probe image was identical or different than the last seen image in the motion.

Probe tasks are well validated in motion perception research. They have been used to measure the motion of both naturalistic [30, 31, 33] and abstract stimuli [29], and how they are affected by sound [84]. In contrast to mouse responses, they more directly probe the perceptual representation of moving stimuli, without drawing upon visuospatial “motor” maps that are used to spatially guide one’s limbs to targets in the environment [36, 57, 58]. Importantly, the non-spatial nature of the required button presses renders them unaffected by the biases acting on mouse judgments and allows us to directly measure the predictive component of robot motion perception in Experiments 1a and 1b. We should therefore find that participants are more likely to identify probe stimuli further along the trajectory with the hand’s last seen location, compared to probe stimuli in a previous part of the motion. Moreover, if sounds are integrated with the perception of the motion, these mis-localisations should increase when the motions are accompanied by longer sounds than when accompanied by shorter sounds.

Hypothesis: We predict that participants will misidentify probe stimuli further along the trajectory as the robot hand’s last seen location, compared to probe stimuli in a previous part of the motion. Moreover, these mislocalisations should increase when the motions are accompanied by longer sounds than when accompanied by shorter sounds if the sounds are integrated with the perception of the motion.

### Methodology

#### Participants

Participants in Experiment 2a were recruited through the University of Aberdeen’s research participation scheme (24 participants, 19 women including trans women, 5 men including trans men, mean age 24.1, SD = 6.91, 21 right-handed). In Experiment 2b, recruitment occurred through the participant recruitment platform Testable minds (32 participants, 22 men including trans men, 10 women including trans women, mean age 31.9 years, SD = 8.09, 29 right-handed). Participants provided electronic informed consent as part of the experimental briefing and were reimbursed with course credits or $\pounds$ 7.20 respectively. Each experiment took approximately 45 min.

The final sample of Experiment 2a, 19, provides .9 power to detect effect sizes of $d =.787$ $(n_{p}^{2} = 0.357)$. For Experiment 2b, the final sample of 26 provides .95 power to detect effect sizes of $d =.736$ $(n_{p}^{2} = 0.360)$.

#### Procedure

No adaptions were made to the apparatus or stimuli (Sect. 2.1.3) in the transition from Experiments 1b to Experiments 2a and 2b.

**Fig. 3:**
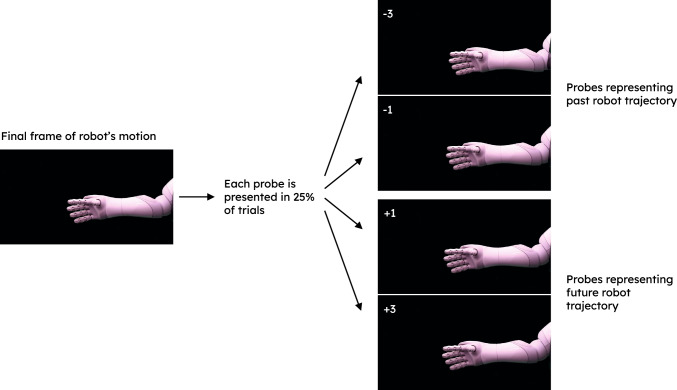
Possible static probes that participants selected to be the ’same’ or ’different’ to the final frame of the action sequence

Participants first gave informed consent and received experimental instruction. Afterwards, eight training trials (identical to the experimental trials) were completed. These training trials, and the instructions preceding then, could be repeated if the participant wished. The experiment consisted of 288 trials, presented in six blocks of 48 trials each. Within each block, conditions were presented randomly, and counterbalanced such that each combination of the two action directions, two sound offsets and three action lengths were presented equal number of times. Between each experimental block there were two-minute breaks, with a countdown indicating the rest time remaining, and re-displaying the experimental instructions. At the end of the experiment, as in Experiment 1b, participants were asked to make a free-text guess of the experimental hypothesis, and were given a funnel debrief, investigating their ability to detect the sound manipulation (see Supplementary Material, testing for demand effects).

As in Experiment 1a and 1b, at the start of each trial participants were presented with a static image of a robot’s hand. Following a randomly generated delay between 1000 and 2500 ms the robot would either reach to the left, or withdraw to the right. Synchronously, the robot’s sound would start to play as it moved across the participants’ screen. As in Experiment 1b, the robot stepped through the action sequence by three, four or five frames. The last frame was immediately replaced by a black screen. The robot’s sound either stopped 100 ms before the robot finished moving, or 100 ms after its motion terminated. After a blank screen of 430 ms, participants were then presented with a static probe image of the robot’s hand and asked to judge if the displayed probe was ‘different’ to the last position of the robot’s hand. The presented static probe could show the hand either three frames behind its final position, one frame behind, one frame ahead, or three frames ahead, relative to the robot’s direction of motion (see Fig. 3).

‘Different’ responses were recorded by the participant pressing the ‘spacebar’ of their personal device. Participants were asked to signal “same” responses—that they perceived the presented probe hand to be in the same position as the hand’s last position before the black screen—by not pressing a key. In Experiment 2b, auditory feedback (a simple chime) was given when the participants pressed the spacebar, to let them know that their response had been recorded. Participants had a maximum of 5 s to make their judgement. The next trial began 5 s after the probe response stimulus was displayed, independent of participant response.

#### Analysis

The dataset was pre-processed using R [65]. Data from participants who did not finish the experiment were excluded as well as the data from participants who met one of the exclusion criteria (see 3.1.4). Motion Overestimation, expressed as the weighted means of the proportion of participants ‘same’ response, was calculated for each participant for each condition. Statistical analysis was completed using the ‘ezANOVA’ function from the ‘ez’ [40] library.

While biases in perception using similar probe tasks have been quantified in various ways (see [28], for overview), we used the weighted means approach [22], as this technique provides a measure that weights forward and backwards probes (future/past) with equal importance, while weighting responses that are further in the past, or future of the action sequence more strongly. The weighted means of the proportion of ‘same’ responses per condition was calculated as is represented in Eq. 3.

3 $$\begin{aligned} p = \begin{pmatrix} -3\\ -1\\ 1\\ 3 \end{pmatrix},MO_w = \frac{\sum _{p = -3}^{p = 3}{(\frac{{\bar{x}}_{s,p}}{{\bar{x}}_{T,p}}}) \times p}{\sum {\frac{{\bar{x}}_{s}}{{\bar{x}}_{T}}}} \end{aligned}$$

In which $MO_w$ is the weighted shift in motion overestimation, and positive $MO_w$ indicates forward displacement from the robot’s hand in the direction of motion. Negative $MO_w$ represents displacement against the direction of motion. *p* denotes the probe image presented to participants, with options ($-3, -1, 1, 3$), corresponding to the probe image being three frames into the past of the robot’s action sequence, one frame in the past, one frame in the future, or three frames into the future. ${\bar{x}}_{s,p}$ represents the sample mean of ’same’ responses at a single probe, while ${\bar{x}}_{s}$, the total mean of same responses over all probes. ${\bar{x}}_{T,p}$ denotes the mean of the total responses (’same’ plus ’different’) at a single probe, and ${\bar{x}}_{T}$ the total responses across all probes.

#### Exclusion Criteria

Participants were excluded if their mean proportion of ‘same’ responses was less than 10% or greater than 90%. This was the case for zero participants in Experiment 2a and Experiment 2b. Additionally, participants were excluded if they did not make at least 10% more ‘different’ responses to probe stimuli that were further away from the hand’s last seen location and visually obviously different (three frames further along, or three frames further back) compared to probes that matched the hand’s disappearance point more closely and were barely perceivable (one frame further along the trajectory or one frame further back). The absence of such a difference would indicate an insensitivity to even the largest differences between hand disappearance points and probe stimuli, and imply a general lack of attention, visual acuity, or a general misunderstanding of the task. These exclusion criteria were decided a priori based on pilot data (Experiment 2a), and preregistered before collecting data for Experiment 2b. 5 participants in Experiment 2a, and 6 participants in Experiment 2b, were excluded for this reason. The final sample had the following demographics: Experiment 3: 19 participants, 16 women including trans women, 3 men including trans men, mean age 23.8, SD = 6.43, 16 right-handed; Experiment 4: 26 participants, 18 men including trans men, 8 women including trans women, mean age 32.8 years, SD = 8.28, 23 right-handed. It should be noted that all preregistered findings for Experiment 2a and 2b remain significant if the exclusion criteria are not applied.

### Results: Experiments 2a and 2b

Each participant’s weighted mean motion overestimation scores were entered into a $2 \times 2 \times 3$ repeated measures analysis of variance (ANOVA) with factors Action Direction (Reach vs. Withdraw), Sound ($-100$ ms vs. $+100$ ms) and End Position of motion termination relative to the centre of the participant’ screen on the x-axis (Centre, Middle, Outer).

**Fig. 4:**
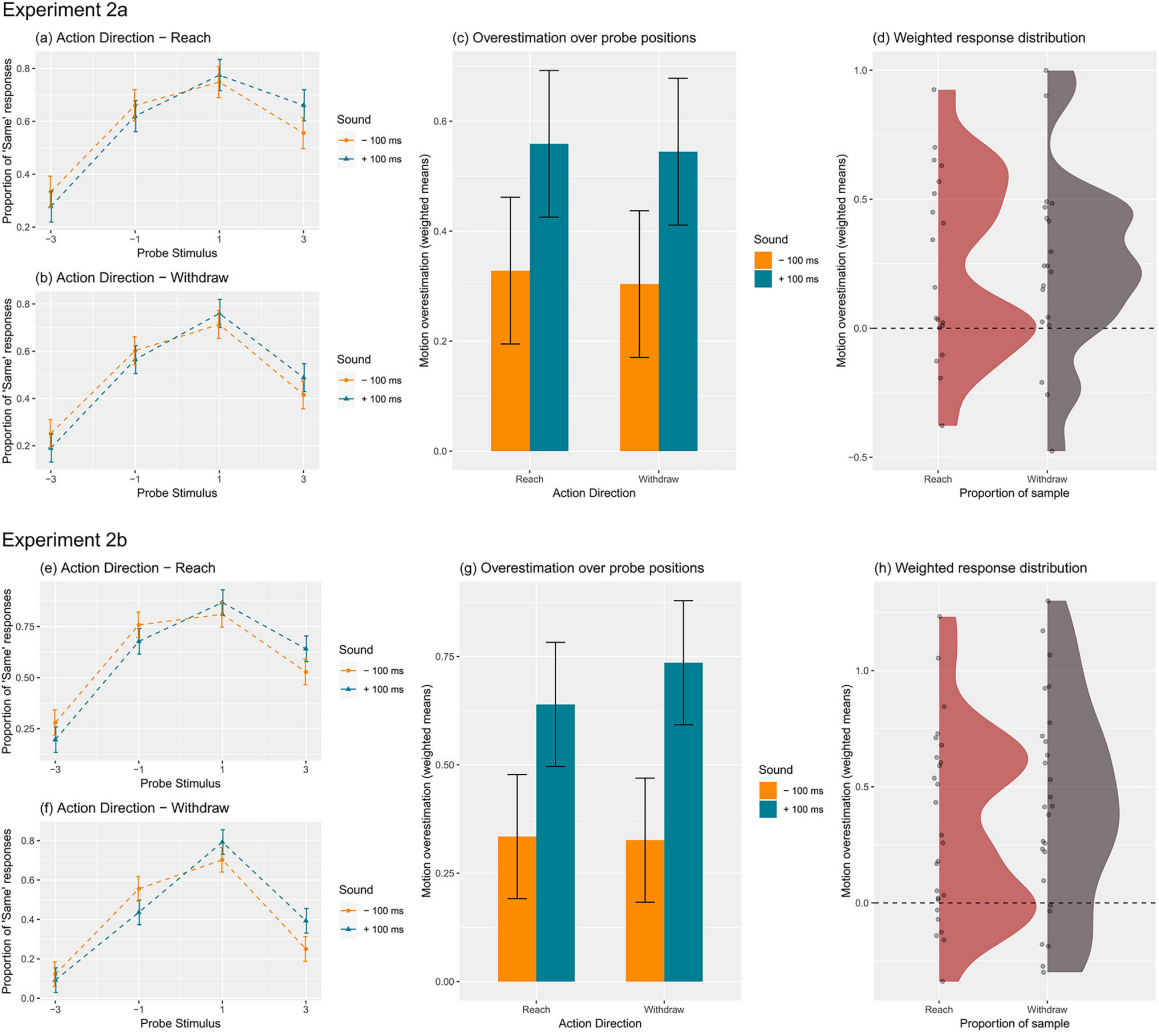
For Experiment 2a, **a** and **b** show the proportion of trials in which the participant judged the probe position to be the same as the final position of the robot is plotted at each level of the probe stimulus ($-3, -1, 1, 3$) for Reaches and Withdraws, respectively. In **c** motion overestimation is expressed as a weighted mean. In **d**, each dot represents each participant’s difference in their weighted mean motion overestimation scores between the longer sound and shorter sound conditions, while the cloud presents the density of the scores for the sample. Error bars in **a**, **b** and **c** represent 95% confidence intervals for the main effect of Sound. For Experiment 2b, the same is shown via (**e**), (**f**), (**g**) and (**h**)

As preregistered for Experiment 2b our hypothesis is that participants will generally overestimate the robot’s motion, particularly during the longer sound duration, compared to the shorter sound duration. General overestimation—that participants’ perception is biased towards their prediction of the future location of the robot hand—is characterised by the intercept term in our ANOVA model, with generally positive values indicating over-estimation of motion towards the predicted next steps and negative values indicating under-estimation. The crucial hypothesis that longer robot sounds will prompt participants to perceive the hand further into its future trajectory than shorter sounds is represented by the main effect of Sound.

As with Experiments 1a and 1b, since we have no additional predictions, all other main effects and interactions are treated as incidental unless they meet the adjusted alpha threshold of .007 to correct for incidental findings in multi-factor ANOVAs [13].

#### Experiment 2a

The analysis of motion overestimation ($MO_w$) revealed the predicted effect that participants’ perceptual responses were biased towards probes representing the robot’s future trajectory, as opposed to probes representing the recent past, indicated by a significant intercept $(F(1, 18) = 16.6, p <.001, n_{p}^{2} =.480)$. For both reaches [Fig. 4(a), and withdrawals Fig. 4(b)], participants were therefore more likely to identify probes in a future location as “same” than probes in a past location, consistent with the general overestimation of perceived motion into its predicted future location (representational momentum [27, 30, 49].

Importantly, our main prediction—that robot sounds that terminated after the shown motion would increase this bias towards identifying future probe locations as “same”—was confirmed by the main effect of Sound $(F(1,18) = 15.4, p <.001, n_{p}^{2} = 0.461)$, indicating larger overestimations for longer than shorter sounds [see Fig. 4(c)].

In addition to these predicted findings, the ANOVA also revealed an interaction between Action Direction and End Position $(F(2, 36) = 8.77, p <.001, n_{p}^{2} = 0.326)$, which is independent of our main hypotheses. It reflects that overestimation is more pronounced for the shorter than the longer movement durations.

Our ANOVA model indicated no additional main effects or interactions that met the adjusted threshold ($F < 4.99, p > 0.012$).

#### Experiment 2b

Experiment 2b successfully replicated all relevant findings of Experiment 2a. The ANOVA first indicated a significant intercept $(F(1,25) = 62.5, p <.001, n_{p}^{2} = 0.714)$. As in Experiment 2a, participants’ responses were generally biased towards probes representing the future steps of the robot motion kinematics [Fig. 4 (e), (f) and (g)]. Moreover, as in Experiment 2a, the ANOVA revealed the crucial main effect of Sound $(F(1,25) = 28.7, p <.001, n_{p}^{2} = 0.534)$, confirming that longer sounds increased the overestimation of perceived motion relative to shorter sounds [reflected in Fig. 4 (e), (f) and (g)].

Next to these predicted results, Experiment 2b also replicated the interaction between Action Direction and End Position, $(F(2, 50) = 6.44, p =.004, n_{p}^{2} = 0.205)$, plus a main effect of End Position $(F(2,50) = 6.21, p =.004, n_{p}^{2} = 0.20)$. This again reflects that motion overestimation is more pronounced for the motions ending in central locations (Centre), and weaker for motions ending in the periphery (Middle and Outer) motions, specifically for withdrawals relative to reaches.

Our ANOVA model indicated no additional main effects or interactions that met the adjusted threshold ($F < 1.65, p > 0.203$).

## Supplementary Information

### Experiment 1: y-axis

Since the actions that the robot performed were leftward reaches or rightward withdrawals, we expected perceptual biases to be most dominant on the x-axis. However there is a small motion variation on the y-axis that could induce the same effects as we find on the x-axis. We report findings from the y-axis, using the identical analysis to Experiment 1a and 1b on the x-axis in the Supplementary Information—Experiment 1 y-axis), which broadly replicates the findings from the x-axis.

### Learning Effects

The current analysis does not allow us to measure possible changes in our findings over the course of the experiment, such as possible learning effects. Understanding if the perceptual changes we report remain stable throughout an extended human–robot interaction, or if after numerous repetitions of the robot’s actions and sounds the perceptual biases diminish—as the participant learns features of the robot’s motion, is crucial for determining use-cases. To test this, we constructed mixed-linear models to track how the perceptual biases in our replication studies (Experiment 1b and 2b) change over the course of the full experiments. In both experiments, the effect of sound on perceptual judgements was remarkably stable, regardless of how far participants had progressed through the experiments and the number of repetitions they had seen of the different combinations of robot action and sounds. The same was true for the general overestimation of motion in Experiment 2b, which if anything increased slightly over the course of the experiment. Full analysis and discussion can be found in the Supplementary Information—Learning Effects.

### Demand Effects

We conducted additional analysis to ensure the sound-induced perceptual biases observed in the present experiments do not reflect demand effects when participants guessed the experimental hypothesis during the experiment and modified their responses accordingly. At the end of Experiment 1b, 2a, and 2b, participants were (1) asked to make a free text guess of the hypotheses they though we were testing. They were then given a tiered funnelled questionnaire asking (1) whether they noticed an experimental difference between trials other than the direction of the robot’s action, and, if yes, (2) whether they could identify what this change was. It was then (3) revealed that the sound of the robot was manipulated and they were asked to identify which characteristic of the sound changed. These responses were blindly and independently rated by JC and PB in terms of how closely they captured the real experimental hypotheses and sound manipulation. The majority of participants did not identify the hypothesis, or detect a difference in the robot’s sound. Moreover, there was little indication that participants who (1) guessed the experimental hypothesis, or (2) identified the difference in the robot’s sound were any less affected than participants who could not. Full details can be found in the Supplementary Information—Controlling for Demand Effects.

## General Discussion

For productive human–robot interaction, it is crucial that human partners can represent the robot’s motions effectively, so that they can plan their own behaviour in response. Here, we present a task—based on well-established representational momentum designs [19, 28] in experimental psychology—that can measure how people visuospatially represent the movements they observe. We tested whether the sounds that robots produce induce illusory distortions in how observers represent the lower-level kinematic features of their actions and anticipate their next steps. In four experiments, we showed participants brief clips of a robot reaching forward or backward and asked them to localise the robot hand’s last seen location after it had suddenly disappeared from view, either with a mouse cursor (Experiment 1a and 1b) or by matching it to comparison stimuli presented directly after (Experiment 2a and 2b). We manipulated the sounds that accompanied these motions so that the sounds either terminated before or after movement offset (± 100 ms). According to multisensory cue integration frameworks [16], the sounds should be integrated into the perception of the robot’s movement kinematics [84], so that visually identical motions appear more extended when accompanied by a longer sound and less so when accompanied by a shorter sound.

All experiments confirmed the predicted influences of sound on participants’ perceptual judgments. In Experiment 1a and 1b participants were asked to accurately localise the perceived hand disappearance points with the mouse cursor. They reported that the robot’s motions extended further into space—further forward for reaches, further backward for withdrawals—when accompanied by a sound that extended beyond the motion’s offset compared to a sound that terminated before the offset. This bias in visual judgments was present when participants had reliable prior knowledge about the duration of the motion they would see (i.e., the motion always terminated after 410 ms, Experiment 1a), but increased when the timing of the motion offset was unpredictable (Experiment 1b). Together, these findings show that visual motion and sound are dynamically integrated during robot action observation [16, 84], and induce subtle distortions in how otherwise identical visual motions are perceived. Moreover, consistent with frameworks of Bayesian integration, these influences increase when the motion offset can be less reliably estimated [39, 93].

Experiments 2a and 2b then confirmed that the human representation of robot motion is predictive, like the perception of abstract objects [29] and biological human agents [30]. When participants judged the robot hand’s disappearance point against comparison stimuli, they (mis-)identified disappearance points slightly in the future than they really were—further forward for reaches, further backward for withdrawals—with the hand’s final location. Importantly, these predictive representations of robot motion were again affected by the sound that accompanied the motion. People identified a location even further in the future as the hand’s last seen location when the sound extended beyond the motion than when it was accompanied with a shorter sound.

Together, these findings are in line with multisensory cue integration frameworks [16]. Accordingly, when viewing motion, the brain integrates all available information from the same or other modalities (here: audition), as well as prior expectations about how the motion is most likely to continue. As a consequence, people’s representation of observed motion is not veridical, but biased away from what was really observed, towards the expected next steps in the motion sequence [27, 30, 49], and towards information provided by other channels. Our study identifies the sound that accompanies robot motion—an often ignored design parameter—as a crucial component that shapes the generation of these integrated predictive percepts of robot motion.

It is remarkable that the relatively subtle manipulation of sound timing (of ± 100 ms), which remained undetected by the majority of participants even when explicitly asked about any such changes, can induce biases of large effect size in motion judgments, indicating practical relevance. Moreover, sound duration affected not only the control of one’s own movements in space when guiding them to the robots hand’s disappearance points (mouse localisation judgments, Experiment 1a and 1b). In addition, it affected the more fundamental perceptual representation of the observed motions, which is tested by the probe judgment task (Experiment 2a and 2b). Finally, additional analyses (see Supplemental information) showed that, across participants, the effect of sound on motion overestimation was independent of (1) whether they were able to guess the experimental hypotheses, (2) whether they were aware of the different sound durations, and (3) did not decline over the course of the experiment, despite the large number of action and sound repetitions participants were exposed to (see Supplemental analyses). These findings suggest that participants experience the integration of sound and motion similar to other multisensory illusions [1, 9, 23, 51], which affects the perceptual representation of the robot motions at a relatively early stage of visuo-cognitive processing that is mostly outside observers’ awareness and is therefore largely independent from higher-order cognitive influences (e.g., awareness of biases, training or learning effects over multiple exposures, for similar findings outside HRI, see [12, 72] ).

The current findings go beyond prior work that has shown that the sounds robots produce can affect the psychometric assessment of its higher-level socioemotive characteristics, such as whether people feel “safe” when interacting with it [87], how they rate its quality, or its competence [69]. Ours is the first study to show that a robot’s sound affects the perception of even low-level features of its behaviour, such as the kinematics of its movements and its expected next steps. Establishing an influence on the representation of low-level features is important because low-level visuospatial features are what ultimately informs the planning of human cooperation partners’ own actions in response within the common workspace, for example, when accepting an object from the robot, giving an object to it, or when simply navigating around it [78]. Moreover, the perception of the low-level features—such as the smoothness of a motion, its speed and its extent in space—feeds directly into higher-level judgments of more global aspects of its behaviour (e.g., competence, safety). Indeed, there is evidence that problematic emotional user responses, such as those in troughs of the uncanny valley [56], often originate from mismatches between different low-level features (e.g., mismatching motion and human-like appearance [88]) or between low-level features and higher-level impressions (e.g., when a robot’s non-biological facial motions contrast with its human-like appearance [52]).

The present findings show that the sound that accompanies a robot’s actions is both a crucial issue that needs to be carefully considered in its design, and an important tool that designers and engineers have at their disposal to better integrate its behaviour into workflows spread across artificial and human operators. For applications such as in flexible production lines in which humans and robots work together and share a workspace [41, 74], the accurate representation of motion extent is of direct relevance. Sounds that accompany the onset of such motions, but are not fully aligned with their end are frequent (e.g., because of friction of moving parts, starting up or ramping down of motors). Our findings suggest that such situations are likely to induce distortions to how the robot’s behaviour is represented, and lead to problems with interactions, on both the level of more global evaluations (e.g., in terms of the robot’s predictability, jerkiness, and safety) and online action planning (e.g., where to reach for when accepting a tool from the robot), which are likely to increase when human operator’s attention is split towards another, concurrent task [53]. Moreover, our additional analyses (Supplemental Material) suggest that any sound-induced changes do not only affect initial human–robot interactions, but can persist over many iterations, unless they are addressed by specifically designed feedback or come to the human interaction partner’s awareness through (potentially costly) errors. Our findings provide first insights into the features of a robot’s consequential sound that can mitigate such issues, but which can also be easily leveraged when designing intentional sounds to supplement the robot’s actions, to produce seemingly more or less pronounced motions or to improve their predictability.

On a theoretical and methodological level, the present findings translate fundamental psychophysical work on the integration of sound and motion into an HRI context. Prior psychophysical work outside HRI has shown that the representation of moving stimuli can be affected by the sound that accompanies them [84]. However, these studies used abstract stimuli, with limited spatial extent, and sound was manipulated between longer blocks of trials, giving room for longer-term expectations to affect the results. Our research shows that sound affects motion perception in much more ecological contexts, with dynamic, naturalistic, spatially extended realistic robot parts with shading and colour, and where the sound that accompanies the motions changes dynamically with every trial. The findings therefore show that fundamental principles of human multisensory integration [16] provide an effective framework to understand—and manipulate—how robot behaviour is perceived. These frameworks assume that sensory representations, and the subjective perceptual experiences that result from them, are abstracted in probabilistic terms, in terms of likelihoods attributed to each sensory characteristic. These likelihoods are further constrained by concurrent information from other channels [84, 85], and by prior expectations about the object’s forthcoming behaviour [30], with an increased weighting of such influences when sensory information is uncertain, providing an optimally likely estimation of the relevant feature given all sensory inputs [35]. As long as the observer can infer a causal [15] (or statistical) relationship between both cues, the perception of one should influence that of the other, as seen in both experiments [93].

The present findings provide valuable insights into the general mechanism governing the human perception and prediction of a robot’s actions, demonstrating a major role of auditory characteristics in even visual estimates of robot behaviour. However, several limitations and questions remain targets for future research.

First, while the experimental paradigm relies on video representations of robot avatars, prior research suggests that the biases it measures feed directly into the action planning mechanisms that we use to interact with moving objects and dynamic interaction partners such as robots [58, 60]. However, our experiment only tested a limited range of simple actions (reaches and withdrawals). In the real world, people will be interacting with robots executing a larger variety of often more complex, multi-step actions, each with perhaps less straightforward morphologies. It is important to confirm that the present findings will generalise to such cases. Importantly, our theoretical framework of Bayesian multisensory integration [37] predicts that the role of sound would, if anything, increase in such situations. If a robot’s motion becomes more variable and less predictable, other cues—like the sounds used here—would become more heavily weighted in perceptual estimates and should therefore more strongly affect localisation of the robot’s motion [16]. Indeed, such an increase was observed in the comparison between Experiment 1a and 1b, where the increase in variability of motion endpoints was associated with a stronger effect of the sound manipulation in Experiment 1b.

Second, currently, the influence of individual cognitive traits and personal biases on this phenomenon is unclear. Future work could explore whether there are specific characteristics that could serve as predictors of susceptibility to the illusory changes to observed motion. For example, there is evidence that an individual’s susceptibility to audiovisual illusions depends on their so-called temporal binding window, reflecting how closely stimuli from different modalities have to be related in time to be integrated into one percept [81]. Similarly individuals with difficulties in motion processing or general visual processing may exhibit a heightened reliance on auditory cues. Indeed, when in similar illusions visual information is obscured, sound is relied on more strongly in localisation [1], thus intensifying its observed effect. In contrast, participants who are aware of the potential misalignment of sound and motion, or with specific prior experience, may be affected less. Examining how individual differences contribute to the manifestation of this effect would provide a more comprehensive understanding of the intricate dynamics at play, perhaps leading to more sophisticated and tailored human–robot interactions.

Finally, further research could compare whether different sound features—e.g., quieter compared to more energetic sounds [7], sounds that appear smoother or jerkier and stuttering—induce similar distortions, and whether motion perception is also affected by other visual cues, such as kinematic profiles, robot morphology, and the robot’s social cues. It has been shown for example that robots that obey biologically inspired kinematic trajectories (e.g., the two thirds power law, minimum jerk motion profiles) are easier to teleoperate /remote control [38, 79] and elicit stronger attributions of goal-directness [66]. One promising use case of the findings of this study would is teleoperation. Supplementary sound (such as that designed by Robinson and colleagues [69]) could “sonify” a teleoperated robot’s motion and act as implicit feedback for operators in circumstances where precision is paramount.

## Conclusions

This study demonstrated that sound can be used to systematically affect how a robot’s action kinematics are visuospatially represented. A sound with a positive offset (longer sound) elicited a shift in perception in the direction of motion, while a negative offset (shorter sound) evoked a shift in perception against the direction of motion. The findings show that frameworks of Bayesian multisensory integration can productively be applied to robot–human-interaction research and provide an effective framework to design multimodal interactions of artificial agents (robotic and digital). We see both the robust nature of this effect and its large effect size as reason to exploit this effect in physical robotic systems to improve the perceptibility of robotic agents to humans. This study therefore contributes a foundational experimental design to test the influence of robotic design parameters on human perception, which can be built on in further studies. The Bayesian cue integration framework enables designers to create more naturalistic interactions with designed environments and artificial agents in augmented and virtual reality. We argue that the same framework can be exploited for social robotics, and that the methodology used in this study offers a first example for deriving quantifiable relationship between modifiable design parameters and low-level human perception. These results and methodology could lead to easy-to-implement methods that aid the human representation of the teleoperated robot’s motions. This could accelerate innovation in the field of robot remote operations, ultimately leading to increased operational safety and effectiveness.

## Supplementary Information

Below is the link to the electronic supplementary material.

Supplementary file 1 (pdf 123 KB)

Supplementary file 2 (pdf 265 KB)

Supplementary file 3 (pdf 128 KB)

## Data Availability

The experimental files, analysis scrips and datasets generated during and/or analyzed during the current study are available from the corresponding author’s github repository.
