# Supplementary material for: Sonic Sleight of Hand: Sound Induces Illusory Distortions in the Perception and Prediction of Robot Action
Source: Int J Soc Robot. 2024 Feb 17;17(10):1957–75. doi: 10.1007/s12369-024-01105-5 (PMC12568858; doi:10.1007/s12369-024-01105-5)
Supplement: Supplementary file 1 — (pdf 123 KB) [file 12369_2024_1105_MOESM1_ESM.pdf]

Sonic Sleight of Hand: Sound induces illusory distortions in the perception and prediction of robot action Supplementary Information: Demand-Effects

Joel Currie[1*], Maria Elena Giannaccini[2] and Patric Bach[1]

[1]School of Psychology, University of Aberdeen, St Machar Drive, Aberdeen, AB24 3FX, UK.

[2]School of Engineering, University of Aberdeen, Elphinstone Rd, Aberdeen, AB24 3UE, UK.

*Corresponding author(s). E-mail(s): j.currie.22@abdn.ac.uk; Contributing authors: elena.giannaccini@abdn.ac.uk; patric.bach@abdn.ac.uk;

Keywords: human-robot interaction, social robotics, representational momentum, movement sonification, cue-integration, motion perception.

It is possible that part of the measured perceptual changes seen in the present experiments reflect demand effects, if participants guessed the experimental hypothesis during the experiment and adjusted their responses accordingly. Thus, in Experiment 1b, 2a and 2b, following the experimental trials, participants were asked to make a free text guess of the experimental hypothesis tested by the experiments, and we evaluated how closely their guess matched the actual hypothesis tested in the experiment. To do so, two raters (JC and PB) independently evaluated each statement, blind with respect to which participant it came from, in how accurately the guess captured the hypothesis that longer consequential sounds would more strongly bias responses in the direction of motion than shorter sounds. Hypothesis guesses were scored on a three-point scale ranging from zero (no match to the experimental hypothesis), one (their explanation covers a general area of the hypothesis), or two (their explanation captures the hypothesis).

Across the 42 included participants from Experiment 1b, the hypothesis guessing scores of the two raters were highly correlated, $r = .96$, suggesting excellent agreement. The median hypothesis guessing score was zero, indicating that most participants had no or little insight into the experimental hypothesis, with a mean of ($\overline{x} = 0.48$). Nevertheless, to test whether insight into the hypothesis could drive parts of our effects, we correlated each participant's average hypothesis guessing score with the change in perceptual judgments when longer or shorter sound were heard (i.e., each participants' interaction contrast values for the interaction of Action Direction and Sound). We found a weak correlation, ($r = .306, p = .048$). Importantly, the intercept was highly significant, ($t = 6.166, p < .001$), indicating that even those participants with a hypothesis guessing score of zero (no insight into the hypotheses) were still strongly affected by the sound manipulation. Indeed, when running the same ANOVA design

used in our main analysis of Experiment 1b, only including participants with a hypothesis guessing score of zero, the predicted interaction of Action Direction and Sound remains highly significant $F(1, 27) = 34.9, p < .001, n_p^2 = 0.56$. This analysis therefore reveals at best a weak correlation between participants' guessing of the experimental hypothesis and the measured effects, and all relevant effects in Experiment 1b are robust against this influence.

In a second test, we evaluated whether participants' ability to notice the difference between the sounds predicted their results. Structured as a tiered "funnel" questionnaire, we asked each participant first 'Apart from the robot's motion, do you think something else changed between each of the robot's movements?' with a Boolean yes/no input. If participants answered yes, they were asked 'What do you think this change was?' with a free text response. Finally, they were asked 'The factor that was changed between trials was one characteristic of the sound the robot made. Specifically, what do you think was the sound characteristic that was changed?' also to a free text response. Once again two independent raters judged the participants' responses on a three-point scale. The median Sound Detection score was again zero, with a mean of ($\overline{x} = 0.63$). This indicates that the majority of participants could not detect a difference between the two sound offsets, even if explicitly asked and funnelled towards the correct answer. Correlating this rating to participants' responses revealed no relationship between the magnitude of biases caused by different sound offsets, and the ability of the participants to detect a difference in sound offset, ($r = .13, p = .397$). However, as for the hypothesis guessing scores above, our model revealed a highly significant intercept ($t = 6.13, p < .001$), indicating that even participants who could not consciously report the differences in sound conditions were robustly affected. This analysis therefore confirms that a person's ability to detect the different sound offsets does not influence their responses.

The same analysis was conducted on the results of Experiment 2a and 2b. The results fully replicated these findings and revealed no influence of people's awareness of the sound manipulation and the experimental hypothesis on the results. As before, two raters provided hypothesis guessing scores, and the sound detection scores for each participant in Experiment 2a and 2b. The two raters' scores were again well in agreement, for both hypothesis guessing scores ($r = .83, r = .90$ for Experiment 2a and 2b respectively) and sound detection scores ($r = .90, r = .94$).

We then again correlated these scores with the contrast value for how much each participant's motion overestimation score were affected by the sounds (the contrast value for the main effect of sound in the ANOVA). In order to increase the sensitivity of detecting any relationships and mitigate the issue of unreliable correlation scores with a small number of participants (Hedge et al (2018), Schönbrodt and Perugini (2013)), all tests were run over the pooled participants of Experiments 2a and 2b. To ensure that the findings were not influenced by group differences, we present the results both using the original scores and validate them after standardising the results of each group through z-score conversion.

The median hypothesis guessing scores was again zero, with a mean of ($\overline{x} = 0.467$), showing that the majority of participants could not guess the hypothesis. No significant relationship was found when correlating each participant's average hypothesis guessing score to the extent their overestimation was affected by the sound manipulation, on both the raw scores ($r = .099, p = .518$), and the standardised data ($r = .143, p = .347$). However, a highly significant intercept ($t = 5.07, p < .001$) was identified showing that even those participants with a hypothesis guessing score of zero were robustly affected by the sound manipulation.

The sound detection scores confirmed this result. The median sound detection score was zero, with a mean of ($\overline{x} = 0.489$), showing that most participants could not detect the difference between a sound offset -100 ms/+100 ms. Moreover, no significant relationship was identified when scores were correlated with how much each participant's motion overestimation was affected by the sound manipulation, both when raw scores ($r = 0.075, p = .624$) and standardized scores

were used ($r = .142, p - .351$). Our model, however, again detected a highly significant intercept ($t = 5.257, p < .001$), showing a robust effect of the sound manipulation even in those participants unaware of the sound manipulation.

Together therefore, our analysis of the hypothesis guessing and sound detection responses revealed little evidence for an influence of demand effects in the measured perceptual changes. Instead, it confirmed that the wide majority of participants did not have any insight into the experimental hypothesis and was not aware of the sound manipulation, even if explicitly prompted in a funnel questionnaire. Indeed, in all experiments, the result for the sound manipulation remains robust when such an influence is controlled.

References

Hedge C, Powell G, Sumner P (2018) The reliability paradox: Why robust cognitive tasks do not produce reliable individual differences. Behavior research methods 50:1166–1186

Schönbrodt FD, Perugini M (2013) At what sample size do correlations stabilize? Journal of Research in Personality 47(5):609–612
