# Supplementary material for: Sonic Sleight of Hand: Sound Induces Illusory Distortions in the Perception and Prediction of Robot Action
Source: Int J Soc Robot. 2024 Feb 17;17(10):1957–75. doi: 10.1007/s12369-024-01105-5 (PMC12568858; doi:10.1007/s12369-024-01105-5)
Supplement: Supplementary file 2 — (pdf 265 KB) [file 12369_2024_1105_MOESM2_ESM.pdf]

Sonic Sleight of Hand: Sound induces illusory distortions in the perception and prediction of robot action
Supplementary Information: Experiments 1a and 1b - y-axis

Joel Currie[1*], Maria Elena Giannaccini[2] and Patric Bach[1]

[1]School of Psychology, University of Aberdeen, St Machar Drive, Aberdeen, AB24 3FX, UK.

[2]School of Engineering, University of Aberdeen, Elphinstone Rd, Aberdeen, AB24 3UE, UK.

*Corresponding author(s). E-mail(s): j.currie.22@abdn.ac.uk;
Contributing authors: elena.giannaccini@abdn.ac.uk; patric.bach@abdn.ac.uk;

Keywords: human-robot interaction, social robotics, representational momentum, movement sonification, cue-integration, motion perception.

Due to the limited range of change on the y-axis in the primarily left- and rightwards going robot actions, we did not expect to be able to obtain an effect sound duration on location responses on the y-axis. However, as for the x-axis, such an effect would be indicated by an interaction of Action Direction and Sound, indicating that participants would localise disappearance points of reaches slightly higher than of withdrawals for longer compared to shorter sounds. Additionally, the action trajectories followed a slightly U-shaped curve, with the endpoints of the trajectories for both reaches and withdrawals slightly higher than the starting positions (see Figure 1b). A stronger bias in the motion direction induced by longer compared to shorter sounds could therefore also be indicated by a main effect of Sound, with higher perceived disappearance points induced by longer sounds. As we had no other predictions for shifts in perception on the y-axis, all further tests have been corrected for alpha inflation for incidental findings in a multi-factor ANOVA Cramer et al (2015). Only main effects and interactions that meet a (Bonferroni-adjusted) alpha threshold of .006 are reported.

1 Experiment 1a

In Experiment 1a, the predicted interaction of Action Direction of Sound was not found ($F(1, 44) = 2.96, p = .092, n_p^2 = 0.063$), but the expected data pattern was present numerically, as shown in Figure 1 a and b. The ANOVA did however reveal a main effect of Sound, ($F(1, 44) = 9.76, p < .004, n_p^2 = 0.182$). Robot movements accompanied by longer sounds were misperceived higher on the y-axis than shorter sounds, consistent with a perceptual extrapolation on upwards going trajectories for both reaches and withdrawals.

In addition to these expected effects of sound duration, the ANOVA revealed a main effect of Action Direction, ($F(1, 44) =$

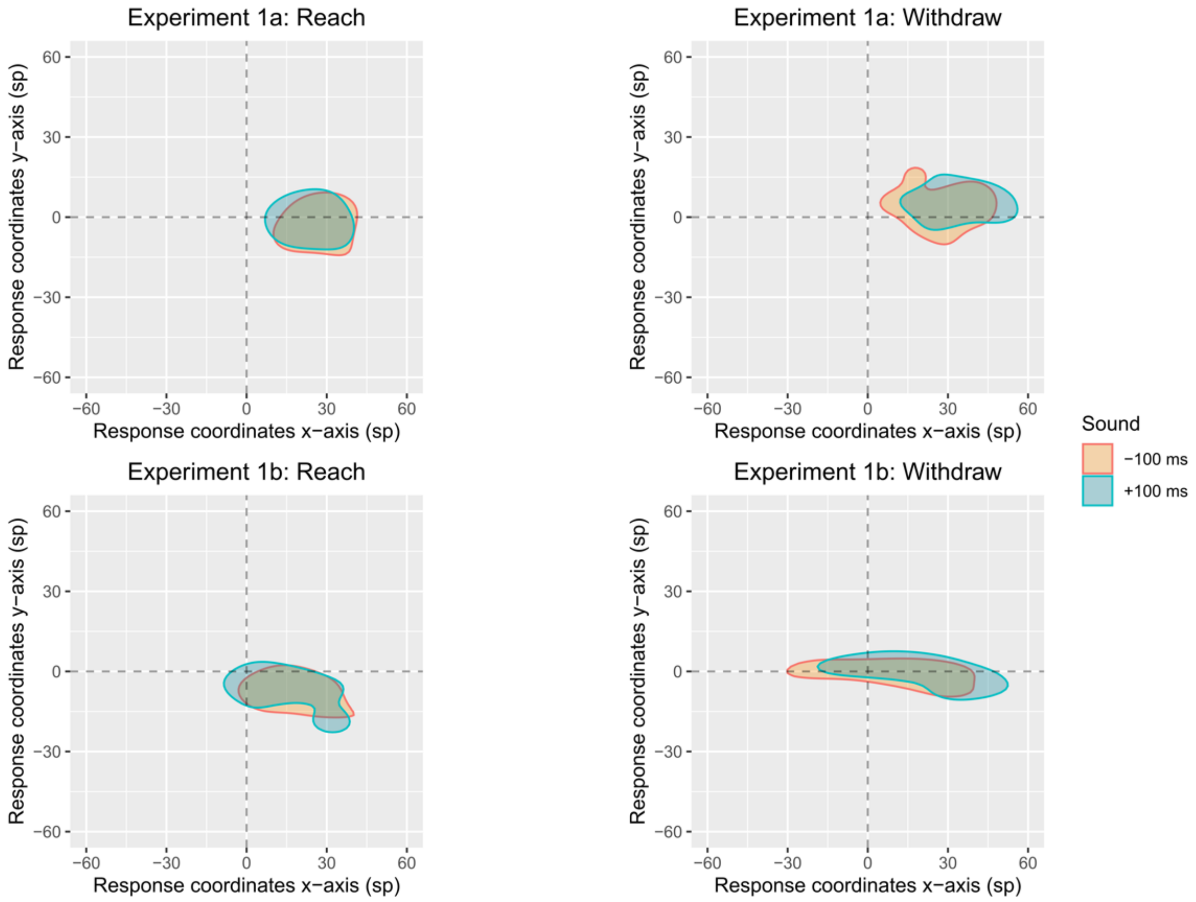

Fig. 1 2D kernel density estimation for spatial distribution of response coordinates expressed as the difference between the real final coordinate of the robot's index finger and participants' response coordinate on the x-axis and y-axis. Response coordinates are shown in universal Scaled Pixels (SP). The (0,0) coordinate represents real final position on any given trial (0 SP difference on each axis). The (0,0) point for reaches has been placed above that for withdrawals as the real final position for reaches was above that of withdrawals.

$43.18, p < .001, n_p^2 = 0.501$) and an interaction of Action Direction and Position, ($F(2, 88) = 30.4, p < .001, n_p^2 = 0.401$). As in the x-axis data, these results reflect the general bias towards of mouse responses towards the centre of the screen, which increases the further along the trajectory the movements terminate.

No other main effects or interactions were found (all $Fs < 4.91, p = .0318$).

2 Experiment 1b

The results of Experiment 1b fully replicate the results of Experiment 1a. As in Experiment 1a, the ANOVA revealed a main effect of Sound, ($F(1, 44) = 22.41, p < .001, n_p^2 = 0.353$), showing that reaches and withdrawals were generally mis-localised further upwards when accompanied by longer sounds than shorter sounds. Moreover, the interaction of Sound and Action Direction that was present only numerically in Experiment 1a was present statistically in Experiment 1b, ($F(1, 41) = 10.26, p < .003, n_p^2 = 0.200$). This confirms that, despite the limited variability on the Y-axis, longer consequential more strongly bias response in the direction of motion – upwards for reaches, downwards for withdrawals – than shorter sounds. Note, however, that while these

findings are entirely consistent with our hypotheses, they were not preregistered and should therefore considered exploratory and interpreted with caution, before being replicated in further studies.

References

Cramer AOJ, van Ravenzwaaij D, Matzke D, et al (2015) Hidden multiplicity in exploratory multiway ANOVA: Prevalence and remedies. Psychonomic Bulletin & Review 23(2):640–647. https://doi.org/10.3758/s13423-015-0913-5
