# Supplementary material for: Sonic Sleight of Hand: Sound Induces Illusory Distortions in the Perception and Prediction of Robot Action
Source: Int J Soc Robot. 2024 Feb 17;17(10):1957–75. doi: 10.1007/s12369-024-01105-5 (PMC12568858; doi:10.1007/s12369-024-01105-5)
Supplement: Supplementary file 3 — (pdf 128 KB) [file 12369_2024_1105_MOESM3_ESM.pdf]

Sonic Sleight of Hand: Sound induces illusory distortions in the perception and prediction of robot action
Supplementary Information: Learning Effects

Joel Currie[1*], Maria Elena Giannaccini[2] and Patric Bach[1]

[1]School of Psychology, University of Aberdeen, St Machar Drive, Aberdeen, AB24 3FX, UK.

[2]School of Engineering, University of Aberdeen, Elphinstone Rd, Aberdeen, AB24 3UE, UK.

*Corresponding author(s). E-mail(s): j.currie.22@abdn.ac.uk;
Contributing authors: elena.giannaccini@abdn.ac.uk; patric.bach@abdn.ac.uk;

Keywords: human-robot interaction, social robotics, representational momentum, movement sonification, cue-integration, motion perception.

The analysis reported in the main manuscript showed that that human observers perceive robot actions as travelling further into the extrapolated future trajectory of the motion, when the sound that accompanies these actions has a longer duration, compared to a sound of a shorter duration. Here, we report additional post-hoc analysis to determine whether participants, over repeated exposures of viewing the same robot actions and hearing the same sounds, would learn features of the action, diminishing the effect of robot sound on motion overestimation.

To address this question, the datasets collected for the replication of both Experiments (Experiment 1b and Experiment 2b) were analysed using a constructed Linear Mixed-Effects Model (MLM) and Generalised Linear Effects Model (GLMM) respectively.

1 Experiment 1b

A MLM was used to validate the findings from our primary analysis and test the effect of sound durations on action overestimation to repeated actions. As in the main analysis, the dependent variable was participants' perceptual shift in each trial, indicating how much more leftward (negative numbers) or rightwards (positive numbers) they reported the hand to have disappeared than it really did. The categorical variables Action Direction and Sound were included as Helmert contrasts and represented the direction ('Withdrawal' [-0.5], 'Reach' [+0.5]) that the robot's hand travelled and whether the sound accompanying this action ended 100 ms before [-0.5] or 100 ms after [+0.5] the motion, respectively. The continuous variable Action Length (3, 4, 5) reflected the amount of frames that were shown of the action before the hand disappeared from view. Finally, Exposure was included as a discrete variable, reflecting the proportion of the experiment that the participant had completed on a

given trial, ranging between 1 and 10. Interactions between Action Direction, Action Length and Sound were permitted. Exposure was permitted to interact with Sound and Action Direction. The random effect's structure that converged with a best fit ($R^2_C = 0.837$) included Action Direction, Action Length and Exposure, each clustered by participant.

As in our primary analysis, an interaction between Action Direction and Sound would indicate that participants' localisation responses were biased by the sound accompanying the robot's action. An interaction between Action Direction, Sound and Exposure would reveal that this effect of sound on action overestimation was sensitive to participants progressing further in the experiment and seeing repeated actions. The main effect of Exposure would signify a general change in the left- or rightwards bias as the experiment progressed. Since this is an exploratory analysis, with multiple factors, excluding the effects of interest only main effects and interactions that reach the adjusted alpha threshold of .005 are reported.

The analysis replicated all findings of the main analysis. The MLM revealed a significant intercept ($E = 20.35, CI = [11.94–28.76], p < .001$), representing the general error rightwards towards the robot hand's centre of mass (see main analysis). An interaction between Action Direction and Action Length ($E = 6.61, CI = [5.01–8.20], p < .001$) represented the increasing "pull" of participants' location judgements towards the centre of the screen the more peripherally the motion terminated. Importantly, as expected, an interaction between Action Direction and Sound ($E = -25.95, CI = [-34.05 - -16.95], p < .001$) was obtained, confirming that, like in the main analysis, longer sounds caused participants to localise hand disappearance points further rightwards for withdrawals and further leftwards for reaches, compared to shorter sounds. The analysis revealed a significant effect of Exposure ($E = -0.85, CI = [-1.39- - 0.31], p = .002$), indicating that participant performance did change over the course of the experiment, reflecting a decreasing bias towards the hand's centre of mass over the course of the experiment. Importantly, there was no interaction between Action Direction, Sound and Exposure ($E = 0.34, CI = [-0.68 - -1.36], p = .515$). Thus, while participants were able to counteract their initial tendency to respond away from the tip of the index finger's disappearance point towards the hand's centre of mass, there was no evidence that the action overestimation induced by the sound was similarly reduced over the course of the experiment.

No further main effects or interactions were indicated by our model that met the adjusted alpha threshold.

2 Experiment 2b

While we have shown that biases in motor responses induced by manipulations in robot sound durations are robust to learning effects in the mouse localisations in Experiment 1b, we wanted to ensure that this was the same for perceptual responses in Experiment 2b. Since responses for Experiment 2b take a binary form (i.e., whether people respond "same" or "different"), we constructed a binomial generalised linear mixed-effects model, with an analogous structure as the MLM used for Experiment 1b.

The dependent variable was participants' response in each trial, with "different" responses coded as 1 and "same" responses coded as 0. The effects structure otherwise mirrored the analysis of Experiment 1b. Two additional factors, Probe and Probe Selection, were included, both representing how many frames in the past/future the probe presented to participants was from the real final frame of motion. Probe was a linear continuous variable (-3, -1, +1, +3), capturing how much further the probe was in the future (positive numbers) or the past (negative numbers) of the last seen action image. In contrast, Probe Selection characterises that participants should be more likely to respond "same" to a probe that is close to the real disappearance point (-1, +1) than to a probe that is further from the real disappearance point (+3, -3). Probe Selection was modelled parabolically.

Action Direction, Action Length, Sound, Probe, Probe Selection and Exposure are included as fixed effects. Interactions between Probe,

Sound and Exposure were present, as well as interactions between Action Direction and Action Length. Random effects, Action Direction, Action Length, Probe, and Exposure were clustered for each participant. Random slopes were not included as when they were present the model did not converge. The model described above was the model of best fit $R^2_C = 0.492$, that converged.

As with Experiment 1b, an effect of Exposure would indicate that participants performance changed over the course of the experiment, showing an increase of "same" or "different" responses overall. A main effect of Probe suggests that the general overestimation into the predicted future of a robot action was still present in the trial-by-trial analysis, while a main effect of Probe Selection that participants were sensitive to the difference between closer and further away probes. An interaction between Probe and Exposure would suggest this effect is sensitive to the repeated viewing of the actions over the experiment. An interaction between Probe and Sound is indicative that the probes chosen by participant are biased by the sound that accompanies the robot's motion, while an interaction between Probe, Sound and Exposure suggests that the effect of the robot's sound on perceptual judgements is affected by viewing the robot's action repeatedly.

Our GLMM model revealed a main effect of Probe Selection ($OR = 1.25, CI = [1.24–1.27], p < .001$), confirming that participants more readily detected outer than inner probes. Importantly, the model also detected a main effect of Probe ($OR = 0.81, CI = [0.73–0.90], p < .001$), revealing the general overestimation of observed motion, with participants being more likely to make "same" responses for probes further in the motion's future. Critically, the interaction of Sound and Probe ($OR = 0.83, CI = [0.79–0.87], p < .001$) was again confirmed, showing, like the main analysis, that motion overestimation increased with longer compared to shorter sounds.

The model did not detect a main effect of Exposure ($OR = 0.96, CI = [0.80 - 1.16], p = .673$), reflecting that the proportion of "different" and "same" responses remained stable as the experiment progressed. However, our model did detect an interaction between Probe and Exposure ($OR = 0.94, CI = [0.90 - 0.98], p = .003$). The model therefore indicates a slight increase in motion overestimation, driven by an increasing acceptance of future probes, and rejection of past probes as the perceived final position of the robot's action. Finally, and most importantly, there was no interaction of Sound, Probe and Exposure ($OR = 1.06, CI = [0.98 - 1.15], p = .154$), indicating that the influence of sound on robot action prediction did not decrease over the course of the experiment.

To control for incidental findings, outwith the main effects and interactions of interest, only additional findings that meet the corrected alpha threshold of $p < .004$ are reported. Our model indicated a main effect of Action Direction ($OR = 0.39, CI = [0.29 - 0.51], p < .001$), and Action Length ($OR = 0.50, CI = [0.37 - 0.69], p < .001$). Both these main effects were present in our weighted means analysis so it was expected that the GLMM would identify them as well.

3 Discussion

Using data from our replication studies (Experiment 1b and 2b), we tested whether any of our effects of interest were sensitive to participants learning features of the robot's actions over repeated viewings (exposures). To accomplish this, linear mixed effects model/generalized linear mixed models were constructed with the same factors used in our primary analysis, with the additional factor Exposure introduced, representing the proportion of the experiment participants had completed at a given trial.

Both models confirmed all aspects reported in the main analysis. In addition, they showed first that – as expected – participants' general performance did change as they progressed through the experiments, so that participants' overall bias away from the finger's actual disappearance point towards the hand's centre of mass in Experiment 1b decreased. Second, and more importantly, both models showed that the general overestimation into the direction of motion was not diminished by seeing multiple repetitions of the same action. If anything, our model of Experiment 2b indicates the general overestimation increases slightly over

the course of the experiment. Third, and finally, the size of the motion over- and underestimation induced by the sound duration manipulation similarly did not change over the course of the experiment. This finding is consistent with the idea that participants experience these biases as subtle perceptual illusion outside their awareness and control. It is also consistent with a locus of the effect in an early phase in visual-cognitive processing that is robust to higher-order cognitive control, suggesting that these perceptual biases are largely resilient to learning or training. The apparent stability of the effect of sound duration on the perception and prediction of robot actions reinforces our proposal for its use in real-world HRI applications.

A limitation of this analysis is that our data only captures participants which have been exposed to 30 or 45 minutes of repeated robot actions (Experiment 1b and 2b respectively). It is possible that over a longer timescale the stability of the observed effect could change. Future work could employ a longitudinal design to eliminate this possibility.
